# *In vitro* and *ex-vivo* evaluation of topical formulations designed to minimize transdermal absorption of Vitamin K1

**DOI:** 10.1371/journal.pone.0204531

**Published:** 2018-10-05

**Authors:** Ramina Nabiee, Barent Dubois, Laura Green, Ajay Sharma, Siu Fun Wong, Hamidreza Montazeri Aliabadi

**Affiliations:** 1 Department of Biomedical and Pharmaceutical Sciences, Chapman University School of Pharmacy, Harry and Diane Rinker Health Science Campus, Irvine, California, United States; 2 Department of Pharmacy Practice, Chapman University School of Pharmacy, Harry and Diane Rinker Health Science Campus, Irvine, California, United States; 3 Center for Targeted Drug Delivery, Chapman University School of Pharmacy, Harry and Diane Rinker Health Science Campus, Irvine, California, United States; Universidade Estadual Paulista Julio de Mesquita Filho, BRAZIL

## Abstract

Topical application of Vitamin K1 has been demonstrated to effectively treat papulopustular skin rash, a serious and frequently encountered side effect of Epidermal Growth Factor Inhibitors (EGFRIs). Systemic absorption of vitamin K1 from skin and the resultant consequence of antagonizing EGFRIs anticancer effects jeopardizes the clinical acceptability of this rather effective treatment. The purpose of the present study was to rationally formulate and evaluate the release rate and transdermal absorption of a wide range of Vitamin K1 dermal preparations with a variety of physiochemical properties. A library of 33 formulations with were compounded and tested for Vitamin K1 permeation using hydrophobic membranes and porcine skin mounted in a Fran diffusion cells. Our results demonstrate the lowest diffusion for water-in-oil emulsions, which also demonstrated a negligible transdermal absorption. The statistical analysis showed a significant correlation between *in vitro* and *ex vivo* results. While viscosity did not have a significant impact on the diffusion or absorption of vitamin K1, an increase in the lipid content was correlated with an increase in transmembrane diffusion (not with transdermal absorption). Overall, formulation design significantly impacts the release rate and transdermal absorption of vitamin K1, and confirms the possibility of minimal systemic distribution of this vitamin for this specific purpose.

## Introduction

Molecularly-targeted anticancer agents have become increasingly significant in the management of many types of cancer in 21^st^ century. This category of anticancer agents mostly target proteins involved in proliferation and/or survival of cancer cells. One of these proteins is epidermal growth factor receptor (EGFR), which is upregulated in different types of cancer, and has been the target for a class of anticancer drugs known as EGFR inhibitors (EGFRIs) [1]. EGFRIs include small molecule drugs (e.g., erlotinib and gefitinib) and monoclonal antibodies specific to this receptor (e.g., cetuximab and panitumumab), and have been used in combination with cytotoxic agents with different degrees of success in various types of cancer, including colon, rectum, pancreas, and lung cancer [2]. A common side-effect of EGFRIs, however, is acneiform eruptions known as papulopustular rash, which is seen in more than 50% of patients (up to 86% for cetuximab), with up to 18% of the patients experiencing a grade 3 reaction, which includes Lesions with symptoms ≥ 50% body surface, accompanied with pain, disfigurement, ulceration or desquamation [3]. This papulopustular rash mostly affects the upper trunk, scalp, and face areas [4]. Dry and itchy skin in 12–16% of patients, and microbial infections in 38–70% are among other cutaneous complications of EGFRI-induced papulopustular rash. Reactions with severity grading at 2 or above may result in dose reduction, treatment interruption or discontinuation. These reactions are also associated with worse quality of life scores, especially in younger patients [5].

While the mechanism of skin toxicity induced by EGFRIs is not completely understood, it has been suggested that the response to EGFRIs and the survival rate correlate with the degree of severity and the onset of the skin toxicity [6], which indicates that a similar mechanism of action could is for therapeutic effect and skin toxicity. More interestingly, the severity of skin toxicity and the onset of the symptoms seem to be independent of the type of EGFRI used, and the correlation of toxicity with efficiency of inhibition of EGFR has been reported for small molecule targeting EGFR, monoclonal antibodies, and combination of both [7]. In 1995, a study in transgenic mice with dominant negative mutation in EGFR showed that lack of EGFR activation leads to interfollicular epidermal keratinocyte hyperplasia and necrosis and disappearance of the follicles, accompanied by strong infiltration by inflammatory cells [8]. In a 2002 study on pharmacodynamics of gefitinib (known as ZD1839 at the time), the drug was detected in skin samples of patients receiving systemic drug, and suppression of EGFR phosphorylation was confirmed in all EGFR-expressing cells [9]. The causal relationship between EGFR inhibition and skin toxicity was further explored in a 2009 review paper that linked the location of the skin rash (most commonly seen in scalp, face, and upper chest) with high density of sebaceous glands, where a higher expression of EGFR has been reported [10].

In this study, we investigated the effect of formulation, including water/lipid content, internal/external phase, surfactant system, and viscosity, on the transdermal absorption of vitamin K1 *ex-vivo*. We hypothesized that the characteristics of the formulation can significantly affect the transdermal absorption of vitamin K1, and our objective was to formulate a topical product that is capable of limiting biodistribution of vitamin K1 to the skin, and minimize the risk of systemic absorption. This formulation will be further evaluated in healthy volunteers to confirm the in vitro and ex vivo findings reported here.

## Materials and methods

### Materials

Synthetic vitamin K1 (Phytonadione) was purchased as a raw material from Skin Actives Scientific (Gilbert, AZ), with purity of > 98%. Bees wax, cetaryl alcohol, isopropyl myristate, sesame oil, white petrolatum, polyoxyethylene 23 lauryl ether, polyoxyethylene 2 oleyl ether, sorbitan monooleate, polyethylene glycol (PEG) 400 monostearate, carbomer 940, poloxamer 407, butylated hydroxytoluene (BHT), imidurea, methylparaben, propylparaben, and polyethylene glycol were all National Formulary (NF) grade and purchased from Professional Compounding Centers of America (PCCA; Huston, TX). PEG-30 Dipolyhydroxystearate was a gift from Huntsman Performance Products (Woodlands, TX). Labrasol and Tefose 63 were gifts from Gattefosse (Paramus, NJ). The Durapore Membrane Filters (0.22-micron GV; catalogue number: GVWP09050) used for *in vitro* diffusion test were purchased from EDM Millipore (Burlington, MA). The pig skin used for *ex-vivo* transdermal absorption was excised from freshly slaughtered pig heads that were obtained from a local slaughterhouse (Lizzie Custom Processing Slaughter House, 7310 Pine Ave, Chino, CA). 1-Penthanol (99%, extra pure, ACROS Organics), fetal bovine serum (FBS), and Hank’s Balanced Salt Solution (HBSS) were obtained from Fisher Scientific (Carlsbad, CA). Cholesteryl benzoate (Catalogue number: C75802) and anhydrous hexane (95%; catalogue number: 296090) were purchased from Sigma Aldrich (St. Louis, MO). HPLC vials were provided by Thermo Scientific (Waltham, MA).

### Methods

#### Quantification of vitamin K1

A High-Performance Liquid Chromatography (HPLC)-based analysis method, based on USP recommended assay, was used to quantify vitamin K1 in the formulations, and the extracts from receiving phase used for *in vitro* and *ex-vivo* experiments. Vitamin K1 was extracted using hexanes:1-penthanol 199:1 mixture. Internal standard (cholesteryl benzoate; 10 μg/mL) was added to the sample and the mixture of sample and extraction mixture were vortexed, and centrifuged at 12,000 g for 5 minutes. The hexanes:1-pentanol portion was then collected for analysis. The HPLC system consisted of an Ascentis Si 5um L3 (25cm x 4.6mm) column connected to a Prominance-i Shimadzu Analytical HPLC. Then 15 μL of extraction was injected onto the column and eluted at a flow rate of 1.0mL/min room temperature under isocratic conditions with hexanes:1-pentanol as the mobile phase. Vitamin k1 (retention time ≈ 8.2 minutes) and internal standard (retention time ≈ 3 minutes) were analyzed at 254nm. Standard curves were created in concentration range of 10 ng/mL—10 μg/mL, based on the ratio of the area under the curves (AUC; Vitamin K1 AUC / Internal Standard AUC) vs. concentration. A sample peak for the internal standard and different vitamin K1 concentrations is presented in **S1 Fig**. See the **S1 File** for the validation of the analytical method.

#### Topical formulations of vitamin K1

Topical formulations were compounded with the strength of 0.1% vitamin K1. Even though vitamin K1 is not available in United Sates as a topical pharmaceutical product, a vitamin K1 topical cream is manufactured in Slovenia by Pharmadab (with Brand name Reconval K1), which contains 0.1% active ingredient. Also, the clinical studies on the effect of vitamin K1 on EGFRI-induced folliculitis have been conducted with a similar strength [11, 12]. The formulations were categorized in 4 general dosage form categories: a. ointments: semisolids with hydrophobic external phase, including water in oil or W/O products; b. creams: oil in water or O/W semisolids with aqueous external phase; c. lotions: O/W or W/O products with thinner consistency; and gels (**Table 1**). The formulations were designed to introduce different important variables into the product library: a) Composition of the dispersion: The formulation library includes oil in oil dispersions (formulations O1 to 3), water in oil dispersions (formulations O.W/O4 to 12 and L.W/O22 to 24), oil in water dispersions (formulations C.O/W13 to 21 and L.O/W25 to 27), and dispersion of active ingredient in completely aqueous vehicle (formulations G.P28 to 30 and G.C31 to 33). Vitamin K1 is a hydrophobic active ingredient and its dispersion pattern in the formulation would change depending on the external phase, which could be one of the factors affecting the transdermal absorption; b) Lipid content: The degree of chemical compatibility of the active ingredient and the vehicle could affect the release rate, and therefore, the transdermal absorption. Due to hydrophobic nature of vitamin K1, a wide range of lipid contents were included in the formulation library with both ends of spectrum (0–100% lipid) included.; c) Emulsifier: The emulsifying agent(s) could significantly affect the physical characteristics of the topical dispersions, and therefore different options, including individual and combinatorial systems, were explored; and d) Viscosity: According to Fick’s first law of diffusion, the diffusion coefficient, or diffusivity, directly affects the diffusion rate, and therefore, the release rate from the dosage form, and one of the factors affecting diffusion coefficient is the viscosity of the vehicle. Therefore, the lipid content and composition (proportion of the lipid ingredients that are liquid in ambient temperature) was modified in different formulations to manipulate the viscosity of the dosage form.

**Table 1:** Library of formulations studied for dermal delivery of Vitamin K1.

Dosage form	Formulation Code	Surfactant system†	Gelling Agent	Lipid content††	Lipid composition‡
Ointment*	O1	-	-	100%	CA/WP/BW
	O2	-	-	100%	WP/CA/BW
	O3	-	-	100%	SO/WP/BW/CA/IM
	O.W/O4‡‡	polyoxyethylene 2 oleyl ether	-	75%	WP/CA/SO/IM
	O.W/O5	sorbitan monooleate / polyoxyethylene 23 lauryl ether	-	75%	WP/CA/SO/IM
	O.W/O6	PEG-30 Dipolyhydroxystearate	-	75%	WP/CA/SO/IM
	O.W/O7	polyoxyethylene 2 oleyl ether	-	67.5%	WP/CA/SO/IM
	O.W/O8	sorbitan monooleate / polyoxyethylene 23 lauryl ether	-	67.5%	WP/CA/SO/IM
	O.W/O9	PEG-30 Dipolyhydroxystearate	-	67.5%	WP/CA/SO/IM
	O.W/O10	polyoxyethylene 2 oleyl ether	-	60%	WP/CA/SO/IM
	O.W/O11	sorbitan monooleate / polyoxyethylene 23 lauryl ether	-	60%	WP/CA/SO/IM
	O.W/O12	PEG-30 Dipolyhydroxystearate	-	60%	WP/CA/SO/IM
Cream**	C.O/W13	polyoxyethylene 23 lauryl ether / polyoxyethylene 2 oleyl ether	-	45%	SO/WP/CA
	C.O/W14	Tefose	-	45%	SO/WP/CA
	C.O/W15	Labrasol	-	45%	SO/WP/CA
	C.O/W16	polyoxyethylene 23 lauryl ether / polyoxyethylene 2 oleyl ether	-	37.5%	WP/SO/CA
	C.O/W17	Tefose	-	37.5%	WP/SO/CA
	C.O/W18	Labrasol	-	37.5%	WP/SO/CA
	C.O/W19	polyoxyethylene 23 lauryl ether / polyoxyethylene 2 oleyl ether	-	30%	WP/CA/SO
	C.O/W20	Tefose	-	30%	WP/CA/SO
	C.O/W21	Labrasol	-	30%	WP/CA/SO
Lotion	L.W/O22	polyoxyethylene 2 oleyl ether	-	74%	SO/WP/CA/IM
	L.W/O23	sorbitan monooleate / polyoxyethylene 23 lauryl ether	-	74%	SO/WP/CA/IM
	L.W/O24	Labrasol	-	74%	SO/WP/CA/IM
	L.O/W25	polyoxyethylene 23 lauryl ether / polyoxyethylene 2 oleyl ether	-	21.5%	SO/CA/WP/IM
	L.O/W26	Tefose	-	21.5%	SO/CA/WP/IM
	L.O/W27	Labrasol	-	21.5%	SO/CA/WP/IM
Gel	G.P28	Labrasol	Poloxamer	0%	-
	G.P29	Tefose	Poloxamer	0%	-
	G.P30	polyoxyethylene 23 lauryl ether / polyoxyethylene 2 oleyl ether	Poloxamer	0%	-
	G.C31	Labrasol	Carbomer	0%	-
	G.C32	Tefose	Carbomer	0%	-
	G.C33	polyoxyethylene 23 lauryl ether / polyoxyethylene 2 oleyl ether	Carbomer	0%	-

†: The total amount of the surfactant(s) was kept constant among all formulations, regardless of the composition of emulsion and aqueous/lipid ratio.

††: The lipid content is calculated solely based on the ratio of the weight of the lipid ingredients added to create the structure of dosage form and the total weight of the formulation. The emulsifier(s), antioxidant, preservatives, and active ingredient were not included in the calculations as “lipids”.

‡: The order of the ingredients indicate the percentage of the ingredients included from the highest to lowest percentage.

*: According to USP definition (a semisolid dosage form with hydrocarbon external phase)

‡‡: The naming convention is based on the dosage form, and the structure of emulsion (e.g., O.W/O, indicates ointment, water/oil) or gelling agent (e.g., G.C, indicates gel, Carbomer). The numbers are based on the order of dosage form in the library.

**: According to USP definition (a semisolid dosage form with aqueous external phase)

**BW**: Bees Wax; **CA**: Cetaryl Alcohol; **IM**: Isopropyl Myristate; **SO**: Sesame Oil; **WP**: White petrolatum

The total emulsifier content was 5% in all water in oil and oil in water dispersions. This total percentage for emulsifiers was reduced to 2% in poloxamer gel formulations due to emulsifying characteristics of poloxamer. The total HLB of the emulsifying system was adjusted at approximately 5 for W/O, and 12 for O/W emulsions. The ratio of emulsifying agents in the combinatorial systems were calculated based on the following equation:

Total HLB = [(Proportion of Surfactant A) × (HLB of Surfactant A)] + [(Proportion of Surfactant B) × (HLB of Surfactant B)]

All formulations were preserved and contained an antioxidant. For oil in oil dispersions (formulations O1 to O3), the excipients were melted and mixed, and after cooling down, vitamin K1 was added and mixed with a homogenizer (Turrax T25, IKA; Medisca, Las Vegas, NV). The final products were passed through an ointment mill (EXAKT 50EC+; PCCA, Huston, TX). For water in oil dispersions (formulations O.W/O4 to 12 and L.O/W25 to 27), vitamin K1 was added to the cooled down mixture of melted lipids, before the aqueous phase was added to the mixture while being homogenized. The final products were passed through the mill. A similar protocol was followed for oil in water dispersions, except that the medicated and cooled mixture of melted lipids was added to the aqueous phase. For gel formulations, vitamin K1 was mixed with the emulsifier(s) and added to the aqueous phase, before adding the gelling agent. For carbomer gels, the pH was adjusted to 6–7 with 1 molar NaOH. Poloxamer gels were stored at ambient temperature after completion of compounding procedure.

The viscosity of the randomly selected formulations was determined using a USS-DVT4 Digital Rotary Viscometer at ambient temperature.

#### *In vitro* transmembrane diffusion

A set of 6 Franz cells (PermeGear; Hellertown, PA) was used for *in vitro* diffusion and permeation studies. For transmembrane diffusion experiments, PVDF 0.22 μ membranes were mounted on the Franz cells, and vitamin K1-containing formulations were weighed and placed on the membrane (equivalent to 200 μg active ingredient; in donor chamber). The receiving chamber was filled with approximately 12.5 mL of 30% FBS mixture in HBSS as the receiving phase, and was stirred with a magnetic rotor and maintained at 37°C for the entire experiment time. One milliliter samples were collected from the receiving phase at pre-determined time points of 0.5, 2, 6, 12, and 24 hours, and replaced with same quantity of fresh receiving phase. The vitamin K1 was extracted using the mobile phase, and the amount of vitamin K1 diffused to the receiving phase was quantified using the HPLC method described before.

#### *Ex-vivo* transdermal absorption

The transdermal permeation of vitamin K1 formulations was tested using the cheek skin obtained from an adult pig. The heads of freshly slaughtered pigs were obtained from a local slaughterhouse and immediately transferred to the lab on ice. Full thickness patches of about 2 × 2 inches size of intact skin were cut from the cheek area of the head. The pieces were cleaned of any underlying subcutaneous fat but leaving the epidermis and dermis both intact. The samples were then rinsed with sterile 1 × PBS, and then either used immediately or kept in serum free low glucose DMEM in 4°C for maximum up to 4 days before being used. The skin samples were then mounted between the donor and receiver compartments of the Franz cell, with the stratum corneum side facing the donor compartment and the dermis facing the receiver compartment. The transdermal permeation was evaluated under similar conditions used for transmembrane diffusion, using the same protocol. However, the experiments were conducted for 12 hours (not 24 hours) due to sensitivity of the extracted skin samples to the temperature used for the test.

#### Chemical stability

Samples of formulation L.W/023 were stored in three different conditions: 4°C, away from light; ambient conditions (20–25°C and natural daylight/laboratory lighting); and 37°C and 95% relative humidity (RH), away from light. Samples were collected at pre-determined time points, and the vitamin K1 content was quantified by the described analytical method.

#### Statistical analysis

All data points are presented as mean ± standard deviation (SD). The correlation coefficient was calculated according to Pearson Correlation Coefficient equation, and its significance was determined by t test. The mean transmembrane diffusion and transdermal absorption among four different categories of formulations were compared by one-way ANOVA and Tukey post-hoc test (p < 0.05). The box graphs were produced using Graphpad Prism 7.04.

## Results and discussion

Currently, topical antibiotics and corticosteroids are used to control the symptoms of this adverse reaction; however, considering the long duration of treatment with EFGRIs, extreme caution is recommended due to drug resistance and adverse effects associated with long term use of antibiotics and corticosteroids, respectively [13]. However, due to the potential link between EGFR inhibition and the skin toxicity, EGFR activators and phosphatase inhibitors were investigated to protect the skin from the toxic effect of EGFRIs, and menadione (vitamin K3) was confirmed as a potent EGFR activator in human epidermal keratinocytes in 2006 [14]. In 2008, a topical cream containing 0.1% vitamin K1 (also known as phytomenadione, phytonadione or phylloquinone; a naturally occurring vitamin in certain vegetables) and urea was used on 30 patients after the cutaneous toxicity was documented, and positive response was reported for all patients within 18 days [15]. A pilot clinical trial on prophylactic effects of vitamin K1 cream in patients with metastatic colorectal cancer treated with cetuximab was reported in 2014, which indicated a possible prophylactic benefit for topical vitamin K1 application [11]. Interestingly, a recent placebo-controlled phase II study of vitamin K3 cream showed no benefit for treatment of cetuximab-induced rash [16]. While vitamin K is commonly used in cosmetic products, pharmaceutical topical dosage forms of Vitamin K are not commercially available in United States.

While the selected HPLC analysis method is based on the recommended method in USP/NF [17], the method was validated to ensure reliability of the quantification method. The standard curves consistently showed a linear relationship (r^2^ > 0.99 throughout the project) in the selected concentration range (up to 10 μg/mL). The inter- and intra-day comparisons showed both accuracy and precision in all selected concentrations (**S1 Table**). The CV% was approximately 15 or higher for the smallest selected concentration of 0.2 μg/mL, which was significantly higher than other concentrations, and indicates this concentration as the limit of quantification for this method. Also, a simple extraction method using the mobile phase was used to collect vitamin K1 from the compounded dosage forms and the receiving phase in transmembrane diffusion and transdermal absorption experiments. The efficiency of the extraction method was also explored using this HPLC method compared to the calculated concentration (in triplicate), and a recovery of more than 98% was repeatedly recorded for ointments, creams, gels, and receiving phase.

The compounded dosage forms were controlled for viscosity and the amount of vitamin K1. All compounded products contained the calculated amount of 0.1% w/w within 97.5–102.5%. **Table 2** demonstrates the dynamic viscosity of the selected formulations explored in this study. One formulation was selected from each category. Viscosity of the vehicle is one of the factors that is speculated to affect the diffusion of the active ingredient and the release rate from the dosage form [18, 19]. In this study we manipulated the lipid content and the lipid composition to create a wide range of viscosity among the compounded dosage forms. As expected decreasing the lipid content was associated with a decrease in viscosity. Furthermore, using lipid components with low melting point, which are in liquid form at room temperature, decreased the viscosity significantly, which is evident in comparing the O.W/O5 and L.W/O23 with 75% and 74% lipid contents, respectively, with viscosities of 659 and 357 Pascal-Second, respectively. The poloxamer gel was significantly more viscous than carbomer gel in this study, which is at least partially due to the concentration of the gelling agent used in these groups of formulations. Among the selected formulations, the viscosity of one formulation (O2) was higher than the maximum quantifiable value for the instrument used, which is reported as > 1000 Pascal-Second.

**Table 2:** The dynamic viscosity of selected formulations.

Formulation	Lipid content	Viscosity (Pascal-second; P.S)*
O2	100%	> 1000
O.W/O5	75%	659 ± 33
O.W/O8	67.5%	605 ± 9
O.W/O11	60%	571 ± 18
C.O/W14	45%	501 ± 21
C.O/W17	37.5%	474 ± 11
C.O/W20	30%	412 ± 17
L.W/O23	74%	357 ± 17
L.O/W26	21.5%	103 ± 2
G.P29	0%	993 ± 5
G.C32	0%	213 ± 16

*: The tests were performed in triplicate, and the data is presented as mean ± standard deviation.

For transdermal absorption, the active pharmaceutical ingredient must leave the vehicle, and therefore, diffusion from the dosage form to a receiving phase through a hydrophobic membrane was quantified for all designed formulations. PVDF membranes have been frequently used for this type of *in vitro* evaluation for different therapeutic agents, including 8-methoxypsoralene [20], riboflavin (B₂ vitamin) [21], and doxycycline [22], among others. As a positive control, 200 μg of pure vitamin K1 oil was placed on the membrane to evaluate the transmembrane diffusion of the pure active ingredient (in triplicate). After 24 hours, 98.7 ± 1.1% of the vitamin K1 was detected in the receiving phase, which indicates that vitamin K1 is readily diffused through the membrane.

**Fig 1:**
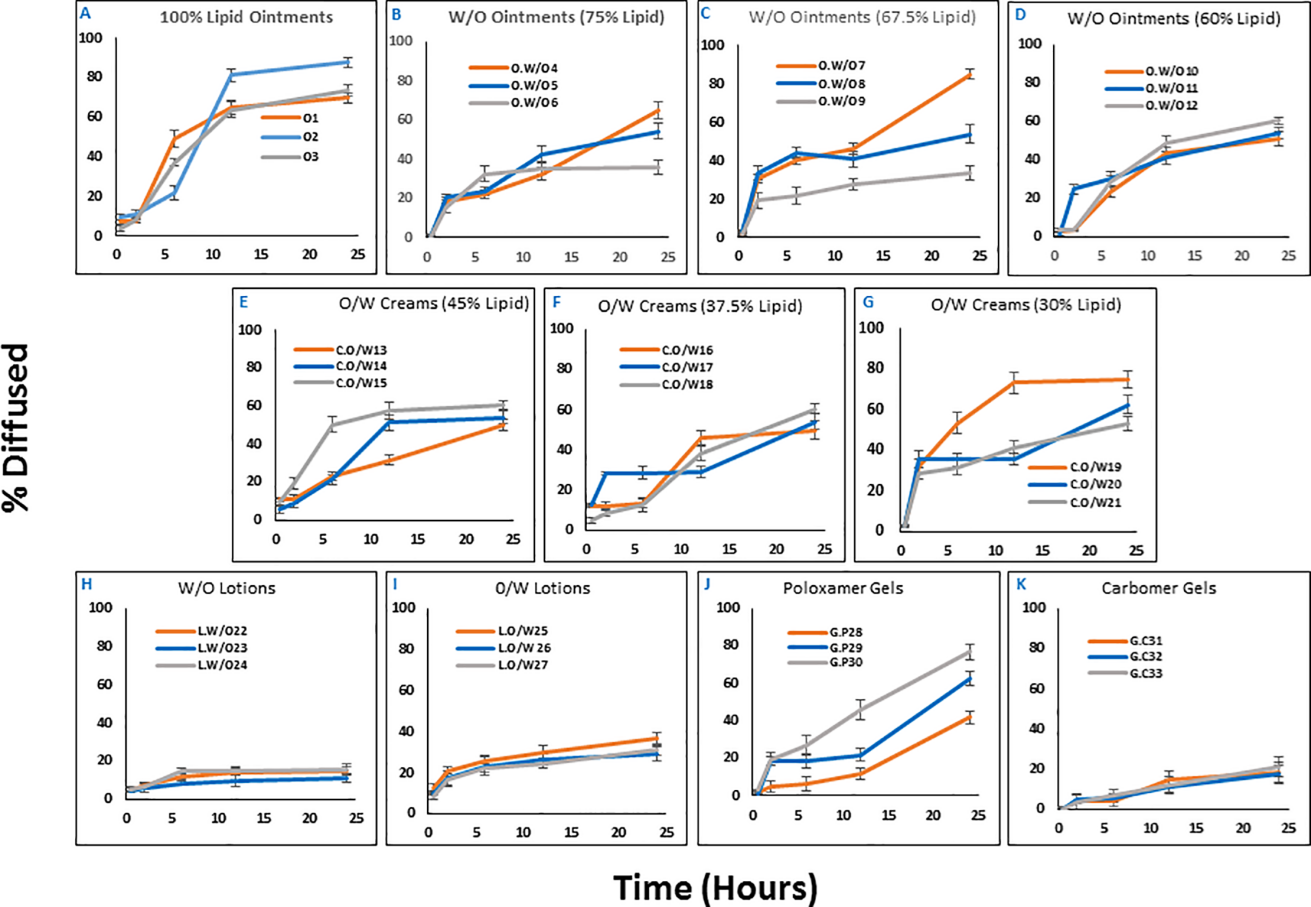
Transmembrane diffusion. The *in vitro* transmembrane diffusion profile of the formulation library using Franz cells. Each panel represent a set of three formulations that shared similar phase composition and lipid content, but differed in surfactant system (n = 3; error bars indicate standard deviation).

The objective of PVDF experiments was to characterize diffusion of vitamin K from our formulations under a simple and controlled setting that offered the advantage of minimal interexperimental variables. This was especially critical for the initial optimization of our formulations. The intent of PVDF diffusion study was not to extrapolate this data for in vivo skin permeation. The results of transmembrane diffusion are summarized in **Fig 1**. At 24-hour time point, formulations with 100% lipid content and no aqueous phase demonstrated the highest diffusion compared to other formulations (**Fig 1A**; Formulation O2 showed the highest cumulative distribution of 87.8%; the only other diffusion over 80% was observed for the W/O ointment formulation O.W/O7 with 85.1%). Overall, formulations in most of the categories demonstrated similar patterns, with lotions and carbomer gels demonstrating the lowest diffusion after 24 hours (~15% for W/O emulsions [**Fig 1H**], ~35% for O/W emulsions [**Fig 1I**], and ~21% for carbomer gels [**Fig 1K**]). The only discrepancy was observed for gels: while poloxamer gels showed relatively high cumulative diffusions (up to 76.8% for G.P30; **Fig 1J**), the carbomer gels showed significantly lower diffusion at the 24-hour end-point. This might be at least partially due to the micelle-forming characteristics of poloxamer, which is a tri-block co-polymer, and has been used for transdermal delivery of different therapeutic agents [23, 24]. Nanoscale emulsions have been studied extensively for transdermal delivery [25, 26], which could partially explain the enhanced diffusion and transdermal absorption observed in this study with poloxamer gels. The surfactant system did not seem to change the diffusion pattern in each category; however, formulations O.W/O4, O.W/O7 (both emulsified with polyoxyethylene 2 oleyl ether), C.O/W19, and G.P30 (both emulsified with combination of polyoxyethylene 2 oleyl ether and polyoxyethylene 23 lauryl ether) showed significantly higher diffusions at the 24-hour end-point compared to similar formulations prepared with other surfactant systems (**Fig 1B, 1C, 1G** and **1J**, respectively). And finally, while most formulations reached a plateau after 12 hours, W/O ointment O.W/O7 and all poloxamer gels showed significant increase in overall diffusion after the 12-hour time point (**Fig 1C and 1J**).

*Ex-vivo* transdermal absorption tests were performed using pigs’ cheek skin. Many reports have indicated structural similarities in pig and human skin including similar thickness, hair follicle content, pigmentation, collagen and lipid composition, healing mechanism [27], and even the flux through the skin and concentrations in the skin (in which regard it is more reliable than commercially available reconstituted skin models) [28]. Similar to transmembrane diffusion test, all formulations included in this study were tested for transdermal absorption. However, due to contamination concerns, the study was only performed up to 12 hours. Also, despite the differences observed in release rates from different formulations, which could create a bias in driving force for transdermal absorption, we decided to keep the concentration of vitamin K1 constant in all formulations. Our objective was to select a formulation for clinical studies that ensures minimum transdermal absorption, and therefore, the selection had to be made among formulations with similar strengths.

**Fig 2:**
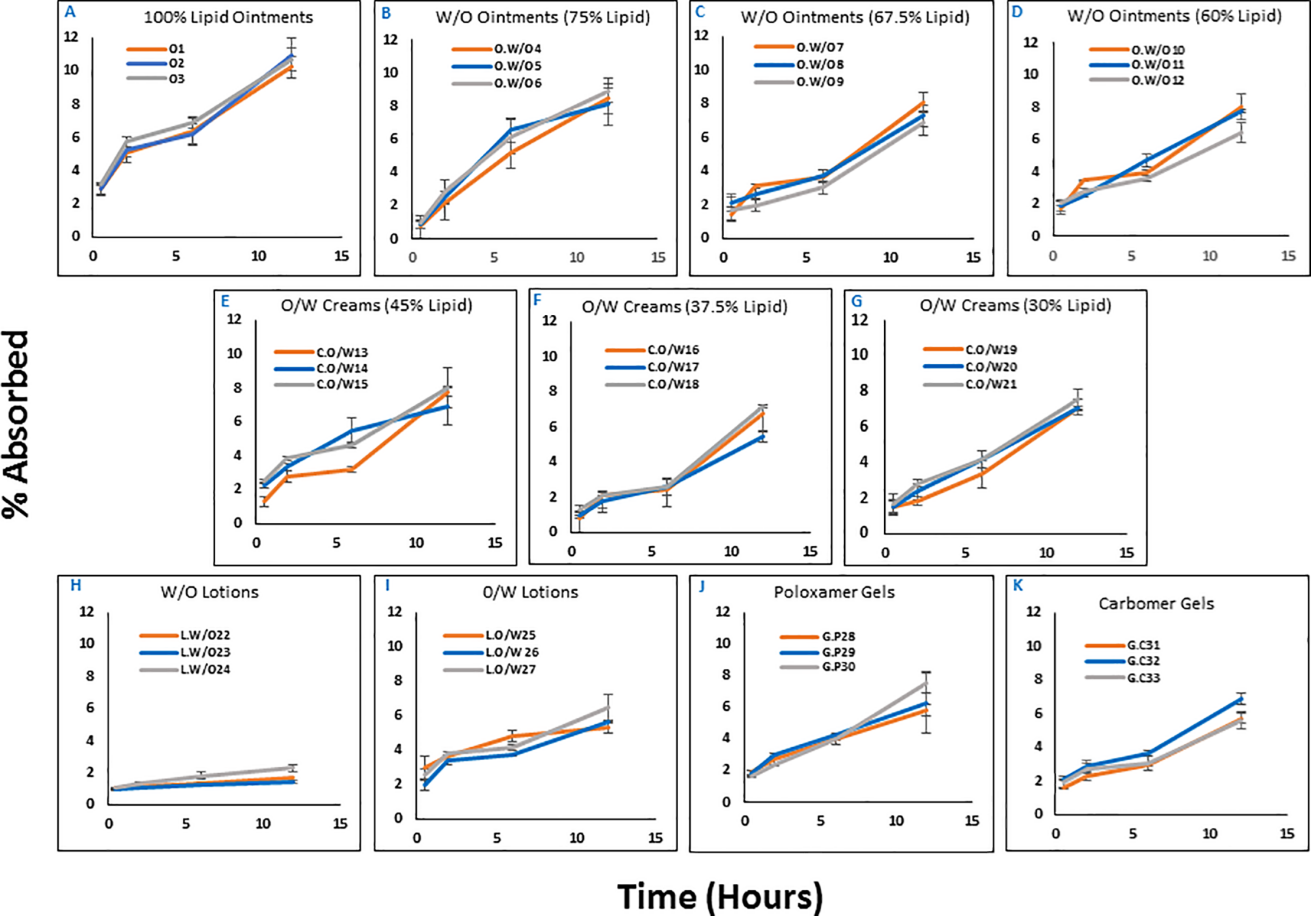
Transdermal absorption. The *ex-vivo* transdermal absorption profile through pig skin for the entire formulation library. Each panel represent a set of three formulations that shared similar phase composition and lipid content, but differed in surfactant system (n = 3; error bars indicate standard deviation).

The results of transdermal absorption for the formulation library is summarized in **Fig 2**. As expected, the overall extent of transdermal absorption was significantly lower than the diffusion at the same time point. Vitamin K1 is a hydrophobic vitamin with a relatively large molecule (MW = 451), and therefore not readily absorbed through intact skin. Also, the variability among different formulations was less significant in this set of studies, which is expected due to the lower overall levels of absorption. However, considering the almost complete transmembrane diffusion of the active ingredient (which indicates sufficient sink conditions and the ability of the receiving phase to solubilize the diffused portions of the active ingredient), the observed variability is most probably due to the formulation composition, and its effect on the release rate of vitamin K1. The overall trend was similar to the transmembrane diffusion pattern. Again, the three formulations with 100% lipid content showed the highest cumulative absorption at 12-hour end-point (up to 10.99% for O2 formulation; **Fig 2A**). Other studies formulations did not reach values higher than 10%. Also, similar to transmembrane experiments, W/O lotions showed the lowest transdermal absorption (1.4% at 12-hour time point for L.W/O23; **Fig 2H**). A similar discrepancy was also observed between the transdermal absorption of W/O and O/W lotions, and poloxamer and carbomer gels; however, the extent of the variability was less significant, as it was mentioned before. The effect of surfactant system on the transdermal absorption was even less significant, and formulations with different surfactant systems did not perform differently in this set of studies. Small variations in this regard did not seem to follow a particular trend.

**Fig 3:**
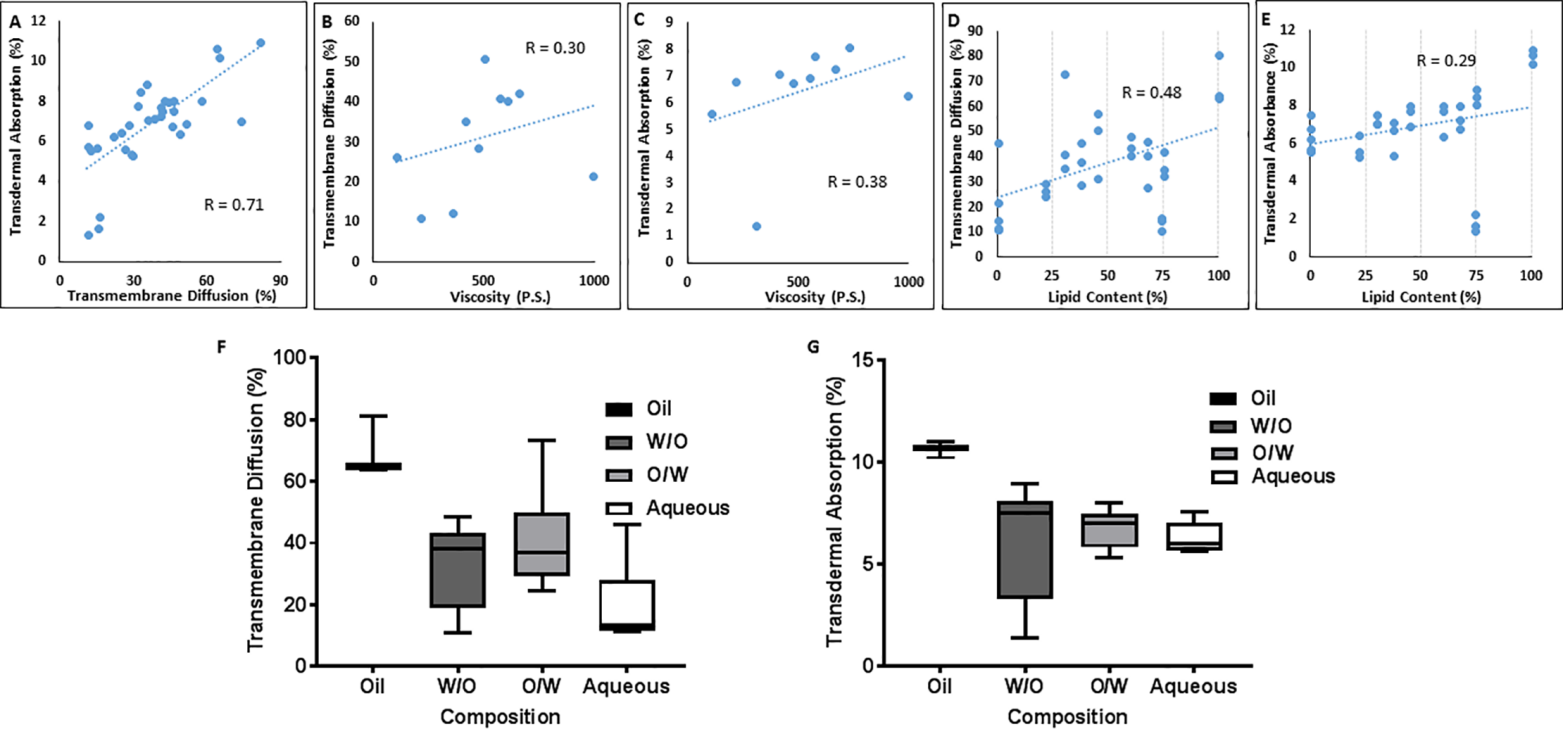
Analysis of data. Statistical analysis of the *in vitro* and *ex-vivo* performance of the formulation library, including the correlation between viscosity and transmembrane diffusion as well as transdermal absorption (A and B, respectively), lipid content and transmembrane diffusion as well as transdermal absorption (C and D, respectively), the extent of transdermal diffusion at 12 hours and the percentage of transdermal absorption at the same time point (E), and a box graph comparison of the mean diffusion and absorption for different category of formulations (F and G, respectively). R represents the correlation coefficient.

**Fig 3** presents the analysis of these two sets of experiments. The transmembrane and transdermal studies showed a significant correlation (R = 0.71, p < 0.001; **Fig 3A**), which indicates that diffusion from the vehicle is the rate-limiting step in the absorption process. This is in line with the near complete transmembrane diffusion of free vitamin K1. Neither diffusion, nor transdermal absorption significantly correlated with Viscosity (**Fig 3B and 3C**, respectively). This is somewhat unexpected, and indicates that the viscosity of the dosage form is not a decisive factor for the transdermal absorption of vitamin K1. While lower viscosity is reported to enhance the transdermal absorption of different drugs (e.g., tenoxicam [18]), it seems that this effect might not necessarily apply to transdermal delivery of every therapeutic agent. The lipid content correlated with transmembrane diffusion (R = 0.48, p < 0.01) but not with transdermal absorption (R = 0.29; **Fig 3D and 3E**, respectively). This might be explained by an enhanced interaction between the vehicle and the hydrophobic membrane with an increase in lipid content. A similar correlation has been reported for the lipophilicity of absorption enhancers and percutaneous permeation [29]. However, this is obviously not the only factor involved as for instance the transmembrane diffusion of O/W creams with 30% lipid content (C.O/W19, 20, and 21) was significantly higher than the W/O lotions with 74% lipid content. In addition to lipid content the effect of formulation composition (all oil, W/O, O/W, and all aqueous) on the diffusion and absorption was also studied (**Fig 3F and 3G**, respectively). One-way ANOVA showed significant difference between groups (p < 0.001), and the ranking of transmembrane diffusion based on Tukey post hoc test was:

Oil>O/W≥W/O>Aqueous

Similar inter-group difference was observed for transdermal absorption (p < 0.01), and the ranking of the groups was:

Oil>O/W≥W/O≥Aqueous

**Fig 4:**
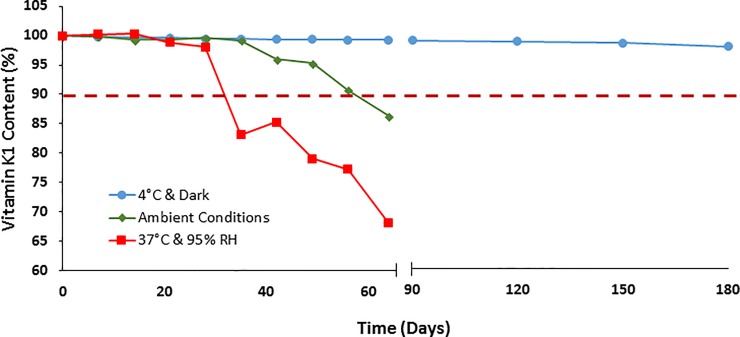
Chemical stability. The phytonadione content in different storage conditions versus time. The red dotted line represents the 90% lower limit, specified by USP for vitamin K1 injectable emulsion.

The formulation with lowest transdermal absorption (L.W/O23) was stored in three different storage conditions to evaluate the chemical stability of phytonadione in this formulation (**Fig 4**). While samples stored in 37°C and 95% humidity, and samples stored in ambient conditions showed a rapid drop in phytonadione content (83.2% vitamin K1 remaining after 35 days, and 90.7% after 56 days, respectively), the samples stored in 4°C and away from light showed expected chemical stability in the studied period (~ 98% remaining after 180 days).

## Conclusion

While transdermal delivery has been an objective for many formulation projects, we studied the possibility of eliminating systemic absorption of vitamin K1 after topical administration (to inhibit the skin toxicity associated with EGFRIs) to minimize the risk of interference with the therapeutic action of these anticancer agents used systemically in cancer treatment. Overall, the W/O lotion formulation L.W/O23 showed the lowest transdermal absorption at 24-hours’ time-point (10.97% diffusion) and the lowest transdermal absorption after 12 hours (1.75% absorption) among the studied groups, and is being evaluated *in vivo* for the dermal delivery of vitamin K1 in healthy volunteers. This formulation could be used concurrently or after onset of therapy with EGFRIs without jeopardizing the patients’ response to cancer treatment.

## Supporting information

S1 Fig HPLC quantification. A sample HPLC graph (A) and corresponding standard curve (B) for Vitamin K1.
(TIF)

S1 File Validation of the analytical method. (PDF)

S1 Table Method validation. The intra- (n = 5) and inter-day (n = 3) values representing accuracy, precision, and bias associated with selected vitamin K1 quantification method (all values presented with three significant figures).
(PDF)
