# Supplementary figures and images for: In vitro and ex-vivo evaluation of topical formulations designed to minimize transdermal absorption of Vitamin K1
Source: PLoS One. 2018 Oct 5;13(10):e0204531. doi: 10.1371/journal.pone.0204531 (PMC6173387; doi:10.1371/journal.pone.0204531)

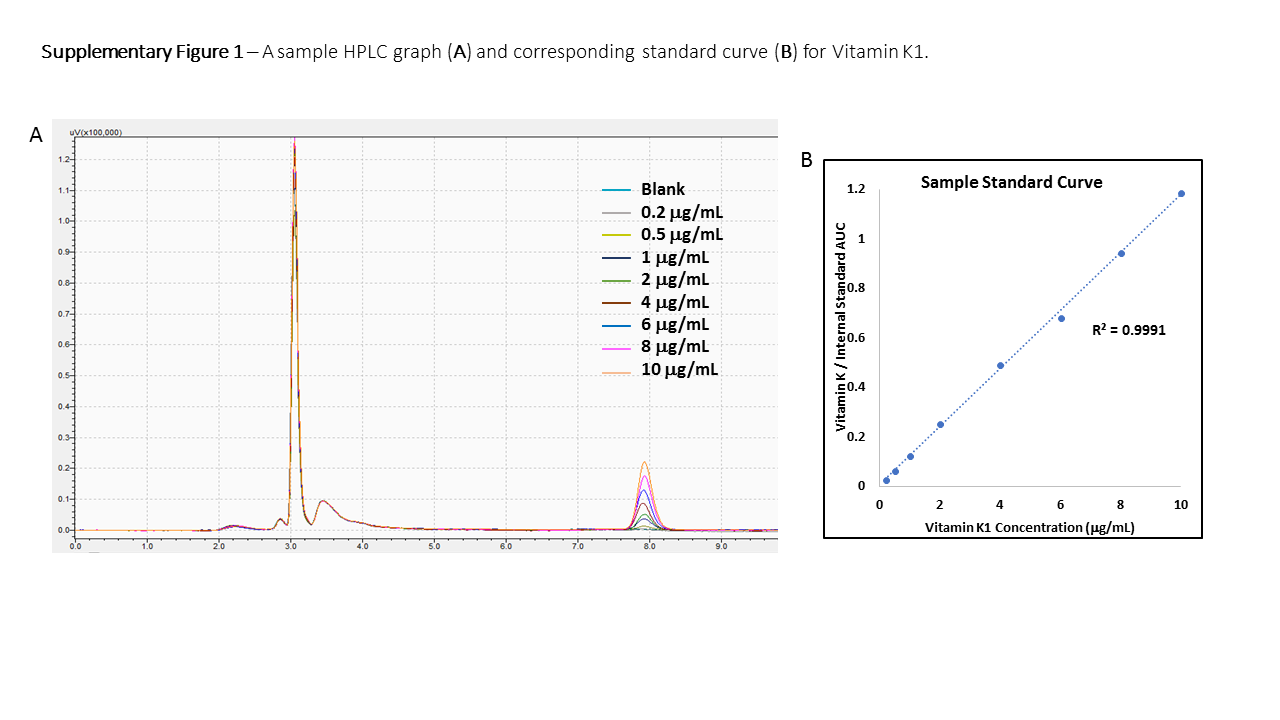

Supplement: S1 Fig — A sample HPLC graph (A) and corresponding standard curve (B) for Vitamin K1. (TIF) [file pone.0204531.s001.tif]
