# Supplementary material for: In vitro and ex-vivo evaluation of topical formulations designed to minimize transdermal absorption of Vitamin K1
Source: PLoS One. 2018 Oct 5;13(10):e0204531. doi: 10.1371/journal.pone.0204531 (PMC6173387; doi:10.1371/journal.pone.0204531)
Supplement: S1 File — (PDF) [file pone.0204531.s002.pdf]

Validation of the analytical method

Standard curves on the same day and different dates were analyzed to validate the method in terms of inter- and intra-day accuracy, precision, and bias by calculating Mean Absolute Error (MAE), Percentage Coefficient of Variation (CV%), and Mean Error percent (ME), respectively [1]. The MAE was determined by the following equations:

$$MAE_{Intraday} = \frac{|Measured\ Concentration\ -\ Nominal\ Concentration|}{Nominal\ Concentration} \times 100$$

$$MAE_{Interday} = \frac{MAE_1 + MAE_2 + \cdots + MAE_n}{Number\ of\ experiments}$$

CV% was calculated based on the following equations:

$$CV\%_{Intraday} = \frac{Standard\ Deviation}{Mean\ Measured\ Concentration} \times 100$$

$$CV\%_{Interday} = \frac{CV\%_1 + CV\%_2 + \cdots + CV\%_n}{Number\ of\ experiments}$$

And, the ME% was determined as:

$$ME\%_{Interday} = \frac{(Measured\ Concentration\ -\ Nominal\ Concentration)}{Nominal\ Concentration} \times 100$$

$$MAE_{Interday} = \frac{MAE_1 + MAE_2 + \cdots + MAE_n}{Number\ of\ experiments}$$

REFERENCE

1. Walther, B.A. and J.L. Moore, *The concepts of bias, precision and accuracy, and their use in testing the performance of species richness estimators, with a literature review of estimator performance.* Ecography, 2005. **28**(6): p. 815-829.
