# Supplementary material for: In vitro and ex-vivo evaluation of topical formulations designed to minimize transdermal absorption of Vitamin K1
Source: PLoS One. 2018 Oct 5;13(10):e0204531. doi: 10.1371/journal.pone.0204531 (PMC6173387; doi:10.1371/journal.pone.0204531)
Supplement: S1 Table — The intra- (n = 5) and inter-day (n = 3) values representing accuracy, precision, and bias associated with selected vitamin K1 quantification method (all values presented with three significant figures). (PDF) [file pone.0204531.s003.pdf]

Table 1. **Method Validation.** The intra- (n = 5) and inter-day (n = 3) values representing accuracy, precision, and bias associated with selected vitamin K1 quantification method (all values presented with three significant figures)

**Nominal Concentration**	**Day 1**	**Day 2**	**Day 3**	**Inter-day**
**0.2 μg/mL**				
Mean Concentration ± SD	0.187 ± 0.0342	0.186 ± 0.0369	0.224 ±0.330	0.199 ± 0.0216
CV%	18.2	19.9	14.7	17.6
MAE	6.28	7.11	12.0	8.45
ME	-6.28	-7.11	12.0	-0.475
**0.5 μg/mL**				
Mean Concentration ± SD	0.471 ± 0.026	0.503 ±0.00340	0.491 ± 0.00868	0.489 ± 0.0162
CV%	5.60	0.675	1.77	2.68
MAE	5.72	0.678	1.73	2.71
ME	-5.72	0.678	-1.73	-2.26
**1 μg/mL**				
Mean Concentration ± SD	1.02 ± 0.0629	1.03 ± 0.0625	0.975 ± 0.0307	1.01 ± 0.0291
CV%	6.15	6.07	3.15	5.13
MAE	2.23	2.85	2.46	2.52
ME	2.23	2.85	-2.46	0.874
**2 μg/mL**				
Mean Concentration ± SD	2.12 ± 0.0806	2.13 ± 0.0547	1.97 ± 0.0483	2.07 ± 0.0913
CV%	3.81	2.56	2.46	2.94
MAE	5.76	6.56	1.72	4.68
ME	5.76	6.56	-1.72	3.54
**4 μg/mL**				
Mean Concentration ± SD	4.22 ± 0.104	4.17 ± 0.0333	3.98 ± 0.0465	4.12 ± 0.128
CV%	2.46	0.800	1.17	1.48
MAE	5.61	4.18	0.524	3.44
ME	5.61	4.18	-0.524	3.09
**6 μg/mL**				
Mean Concentration ± SD	5.83 ± 0.0709	5.78 ± 0.124	6.05 ± 0.0567	5.88 ± 0.142
CV%	1.22	2.15	0.937	1.43
MAE	2.80	3.74	0.754	2.43
ME	-2.80	-3.74	0.754	-1.93
**8 μg/mL**				
Mean Concentration ± SD	7.81 ± 0.136	8.05 ± 0.0886	7.85 ± 0.138	7.90 ± 0.127
CV%	1.74	1.10	1.76	1.53
MAE	2.40	0.575	1.88	1.62
ME	-2.40	0.575	-1.88	-1.23
**10 μg/mL**				
Mean Concentration ± SD	10.25 ± 0.154	10.21 ± 0.0445	9.98 ± 0.0621	10.15 ± 0.149
CV%	1.50	0.44	0.622	0.852
MAE	2.53	2.09	0.243	1.62
ME	2.53	2.09	-0.243	1.46

CV%: Percentage coefficient of variation; **MAE**: Mean absolute error; **ME**: Mean Error; **SD**: Standard Deviation
